# Choline consumption reduces CVD risk via body composition modification

**DOI:** 10.1038/s41598-024-66039-4

**Published:** 2024-07-12

**Authors:** Haomiao Wang, Jinxin Lin, Shitao Fan, Xuyang Zhang, Tengyuan Zhou, Ran Luo, Chao Zhang, Shuixian Zhang, Qingwu Yang, Rong Hu

**Affiliations:** 1 Department of Neurosurgery and Key Laboratory of Neurotrauma, Southwest Hospital, Army Medical University (Third Military Medical University), Chongqing, China; 2 Department of Cardiology, Daping Hospital, Army Medical University (Third Military Medical University), Chongqing, China; 3 Department of Neurology, Xinqiao Hospital and The Second Affiliated Hospital, Army Medical University (Third Military Medical University), Chongqing, China

**Keywords:** Cardiology, Neurology, Risk factors

## Abstract

Despite extensive research on the relationship between choline and cardiovascular disease (CVD), conflicting findings have been reported. We aim to investigate the relationship between choline and CVD. Our analysis screened a retrospective cohort study of 14,663 participants from the National Health and Nutrition Examination Survey conducted between 2013 and 2018. Propensity score matching and restricted cubic splines was used to access the association between choline intake and the risk of CVD. A two-sample Mendelian randomization (MR) analysis was conducted to examine the potential causality. Additionally, sets of single cell RNA-sequencing data were extracted and analyzed, in order to explore the role of choline metabolism pathway in the progression and severity of the CVD and the underlying potential mechanisms involved. The adjusted odds ratios and 95% confidence intervals for stroke were 0.72 (0.53–0.98; p = 0.035) for quartile 3 and 0.54 (0.39–0.75; p < 0.001) for quartile 4. A stratified analysis revealed that the relationship between choline intake and stroke varied among different body mass index and waist circumference groups. The results of MR analysis showed that choline and phosphatidylcholine had a predominantly negative causal effect on fat percentage, fat mass, and fat-free mass, while glycine had opposite effects. Results from bioinformatics analysis revealed that alterations in the choline metabolism pathway following stroke may be associated with the prognosis. Our study indicated that the consumption of an appropriate quantity of choline in the diet may help to protect against CVD and the effect may be choline-mediated, resulting in a healthier body composition. Furthermore, the regulation of the choline metabolism pathway following stroke may be a promising therapeutic target.

## Introduction

Cardiovascular disease (CVD), in particular ischemic heart disease (IHD) and stroke, represents a significant global disease burden in terms of mortality and morbidity. Over the past few decades, there has been a notable rise in the incidence rate, mortality, disability-adjusted life years, and years of life lost attributed to these illnesses and CVD continues to be a major contributor to the global burden of disease^1^.

Among the modifiable risk factors for CVD, dietary risks are considered the most significant behavioral risk factor. In 2017, an estimation revealed that dietary risk factors were responsible for 53% of deaths and 58% of disability related to CVD^2^. A meta-analysis indicated that increased levels of circulating choline are linked to a heightened risk of both CVD and all-cause mortality^3^. While other results from a meta-analysis failed to provide conclusive evidence to support a correlation between dietary choline intake and the incidence and mortality of CVD^4^. Given the contradicting results, the appropriate levelt of daily dietary choline intake is of utmost importance in the primary prevention and to improve the prognosis of CVD.

Choline was first formally recognized as a vital nutrient by the Institute of Medicine (currently recognized as the National Academy of Medicine) in 1998 and is an essential nutrient for human health and plays a crucial role in various stages of life, ranging from embryonic development to adulthood^5^. It plays a pivotal role in the support of perinatal development. Its absence may result in the occurrence of neural tube defects^6^, a collection of serious congenital abnormalities. In preterm infants, choline deficiency is associated with hindered growth of lean body mass, as well as impaired neurocognitive development^7^. Furthermore, inadequate dietary intake of choline has been linked to the development of fatty liver disease, neurodegenerative disorders, reduced bone mineral density, and muscle atrophy^8–11^, indicating that ensuring an adequate daily intake of choline throughout the lifespan is crucial for maintaining health.

The adequate intake (AI) for daily dietary choline was established in 1998 at 550 mg/day for men and 425 mg/day for women, respectively. In 2016, the AI for adults was set at 400 mg/day. These suggestions were developed mainly considering their muscle- and liver-protective effects. However, in the context of CVD, previous studies yielded different findings, and the precise connection between incidence and prognosis of CVD and dietary choline intake has yet to be fully understood, resulting in confusion among researchers. Currently, there are extensive studies focusing on the association between microbiota and CVD. It has been widely established that the negative impact of microbiota on CVD is primarily attributed to the production of trimethylamine-N-oxide (TMAO) from choline, which has been linked to an elevated risk of CVD^12,13^. The association between TMAO and CVD has been established and confirmed by several studies, mediated by the proliferation of foam cells, activation of platelets, disrupted transport of bile acids and cholesterol, and inflammation effect of TMAO^14–17^. However, the elevated level of circulating choline observed in individuals who have experienced a stroke is correlated with better post-stroke cognitive performance^18^, indicating that the tissue-specific metabolic pattern of choline may be associated with divergent prognoses of CVD patients. It is therefore of great importance to identify the optimal dietary intake of choline for the prevention and treatment of CVD and to elucidate the tissue-specific metabolism of choline investigating its potential role in CVD.

In this study, we conducted a comprehensive analysis to explore the associative and causal relationship between dietary choline intake and CVD. A cohort from the National Health and Nutrition Examination Survey (NHANES) from 2013 to 2018 was screened in order to unravel the association between choline intake and the risk of CVD. Then, we examined the dose–response effect and provided a dietary choline intake quantity recommendation based on its CVD-protective effect. Subsequently, Mendelian randomization (MR) was designed with the aim of validating the causality and exploring the mediation factors, such as body composition. Additionally, we investigated the potential effect  of tissue-specific choline metabolism patterns in CVD and the underlying mechanisms employing single-cell RNA sequencing data of brain tissue from stroke models and adipose tissue from different dietary patterns models.

## Subjects and methods

### Study population

The National Health and Nutrition Examination Survey (NHANES) study is a large-scale, nationally representative study of the American population. It was conducted using a multi-stage, stratified approach. The National Center for Health Statistics, a division of the Centers for Disease Control and Prevention in the United States, conducted this assessment to evaluate the nutritional and physical well-being of Americans^19^. Data from three consecutive NHANES with 2-year cycles (2013–2014, 2015–2016, 2017–2018) were collected.

### Definition of exposure and outcomes

All participants had the chance to participate in two 24-h dietary recall interviews. The first recall interview took place at an NHANES Mobile Examination Center, while the follow-up dietary recall interview was conducted over the phone within a 3–10 day period. The daily dietary intake of nutrients and food ingredients from all foods was calculated and recorded in the NHANES database, using the U.S. Department of Agriculture's Food and Nutrition Database for Dietary Studies. The average dietary choline intake from the two 24-h dietary recalls was used for analysis.

The medical condition questionnaire was used to record whether the patients had pre-existing medical conditions. Cardiovascular diseases (CVD) were defined using self-reported history by asking the following question: “Has a doctor or other health professional ever told you that you had a stroke/heart attack/congestive heart failure/coronary heart disease?”.

### Covariates

Variables of interest obtained by questionnaires included basic information of participants on age (years), sex, race, education level, smoking, hypertension, diabetes, body mass index (BMI), waist circumference (WC), fat mass index (FMI), lean body mass index (LMI), left/right arm/leg fat (g), trunk fat (g), total abdominal fat area (cm^2^)/volume (cm^3^)/mass (g), visceral adipose tissue area (cm^2^)/volume (cm^3^)/mass (g), and subcutaneous fat area (cm^2^)/volume (cm^3^)/mass (g).

### Genetic instruments selection of choline metabolites

We systematically searched the UK Biobank dataset for 249 metabolites and identified three choline metabolites for analysis: total choline, glycine, and phosphatidylcholine (N = 114,999). The genetic variants that showed significant associations (p < 5 × 10^−8^) with the three mentioned metabolites were selected as potential genetic instruments for these metabolites. A clumping analysis was conducted to remove genetic variants that showed a correlation with each other (correlation coefficient < 0.001). After a rigorous selection and validation process, 64 genetic instruments were identified for total choline, 61 instruments were identified for phosphatidylcholine, and 49 instruments were identified for glycine.

### GWAS data of stroke and body composition

The  summary statistics genetic data of stroke were obtained from the publicly available database MEGASTROKE consortium launched by International Stroke Genetics Consortium and the details of the data were described in detail elsewhere^20^. Briefly, it is a genome-wide association meta-analysis consisting of 29 genome-wide association studies (GWAS) with 521,612 individuals (40,585 cases and 406,111 controls) of European ancestry. Stroke was defined as rapidly developing signs of focal (or global) disturbance of cerebral function, lasting for more than 24 h or leading to death with no apparent cause other than that of vascular origin. This meta-analysis further divided strokes into ischemic strokes (IS, 34,217 cases). Among all IS cases, 4373 were subclassified as large artery stroke, 7193 as cardioembolic stroke and 5386 as small vessel stroke, according to the Trial of ORG 10172 in Acute Stroke Treatment criteria^20^. The full GWAS summary statistics of the body composition, including lean body mass, appendicular lean body mass, left/right arm/leg fat percentage/mass/fat-free mass, trunk fat percentage/mass/fat-free mass, and whole body fat percentage/mass/fat-free mass were publicly available via the IEU OpenGWAS database^21^.

### Single-cell RNA sequencing data acquisition and processing

Single-cell RNA sequencing data (scRNA-seq) data of the brain in mice with stroke and control mice was obtained from the Gene Expression Omnibus (GEO) database with the accession number GSE167593^22^. The study included three groups: healthy control, ischemic stroke, and hemorrhagic stroke. scRNA-seq data from epididymal adipose tissue was acquired from GSE160729^23^, encompassing both high-fat-diet (HFD) fed mice and low-fat-diet (LFD) fed mice. Seurat's standard process were employed during the analysis. In addition, to analyze the expression of choline metabolism gene signature for each cell, we used UCell^24^ and AUCell^25^ to visualize and quantify gene expression levels. Briefly, UCell and AUCell are two methods used for scoring pathways in gene expression analysis. UCell scores pathways based on the Mann–Whitney U statistic, while AUCell scores pathways based on gene set enrichment analysis using the area under the curve (AUC) value for the selected gene signature.

### Gene ontology enrichment analysis

Gene ontology (GO) analysis is a widely employed and effective approach for gene annotation, as well as for elucidating the biological attributes of large-scale genome or transcriptome data^26^. DEGs were analyzed using the Database for Annotation Visualization and Integrated Discovery (DAVID, https://david.ncifcrf.gov/), an online bioinformatics tool. This tool was utilized to interpret the GO functions as well as to visualize the biological processes (BP), molecular functions (MF), cellular components (CC), and pathways associated with the DEGs^27^.

### Construction of PPI network and module analysis

Protein–protein interaction (PPI) network analysis was performed using Search Tool for the Retrieval of Interacting Genes (STRING)^28^ and the molecular complex detection (MCODE) in Metascape^29^ was used to screen the modules of the PPI network.

### Statistical analysis

The prescribed sample weights were utilized in all analyses. The 2-year sample weight recommended for the period 2013–2018 was employed to compute the updated 6-year sample weights for all participants. Continuous variables in baseline characteristics were analyzed using either Student's t-tests or nonparametric Mann–Whitney U tests. Categorical variables in the baseline characteristics were assessed using Chi-square tests or Fisher's tests. Continuous variables were reported as mean ± standard deviation (SD), whereas categorical variables were presented as numbers (percentage). The propensity score matching (PSM) was employed to pair individuals with and without a history of stroke at a ratio of 1:2. To control for potential confounding variables, including age, sex, race, education, smoking, hypertension, and diabetes. The primary analysis of MR involved the implementation of the random-effect inverse-variance weighted (IVW) method. Sensitivity analysis encompass the examination of heterogeneity and potential pleiotropy, which includes both horizontal and directional pleiotropy, and were assessed by Cochran’s Q statistic and the MR-Egger test (intercept). The intercept derived from MR-Egger regression is widely acknowledged and commonly employed for assessing directional pleiotropy. Meanwhile, the Cochran’s Q statistic obtained from the random-effect IVW approach is considered a useful indicator of heterogeneity, and can be employed to assess potential horizontal pleiotropy. The MR-Pleiotropy Residual Sum and Outlier methods (MR-PRESSO) were employed to evaluate and rectify the presence of horizontal pleiotropy. The analysis were undertaken by the package TwoSampleMR^30^ and MRPRESSO^31^. All data were analyzed using R (version 4.3.1) and p < 0.05 was considered statistically significant.

### Ethics approval and consent to participate

All participants provided informed consent, and the ethics approval was obtained from the research ethics review board at the National Centre for Health Statistics. The NHANES study has received approval from the National Center for Health Statistics Research Ethics Review Board, and all survey participants provided informed consent. No ethical or administrative permission is necessary to access the NHANES database. All methods were performed in accordance with the relevant guidelines. More information can be found online (www.cdc.gov/nchs/nhanes/).

## Results

### Clinical profile of included participants

A total of 14,663 participants aged from 20 to 80 years with the mean age 49 ± 17.52 years who have recorded data of dietary choline intake was included in this study. About 51.9% of the participants consisted of females, 38.0% were non-Hispanic white, 31.3% had some college or AA degree education. In different groups of dietary choline intake, sex, age, race, education level, smoking status, hypertension, and cardiovascular disease (CVD) prevalence rate are significantly different (p < 0.05). The clinical profile of the included participants is shown as quartiles in Table 1.

**Table 1:** Baseline characteristics of the entire study population, both collectively and stratified based on quartiles of dietary choline intake.

Characteristics	Total	Choline Intake Quartile, mg/day				p value
		Quartile1 (< 216.95)	Quartile2 (216.95–303.00)	Quartile3 (303.00–413.60)	Quartile4 (> 413.60)	
Case number	14,663	4034	3511	3511	3607	
Age (SD), year	49.717 (17.517)	49.900 (18.116)	50.628 (17.653)	50.289 (17.320)	48.069 (16.777)	< 0.001
Sex, n (%)						< 0.001
Male	7053 (48.1%)	1234 (30.6%)	1476 (42.0%)	1797 (51.2%)	2546 (70.6%)	
Female	7610 (51.9%)	2800 (69.4%)	2035 (58.0%)	1714 (48.8%)	1061 (29.4%)	
Race, n (%)						< 0.001
Mexican American	2138 (14.6%)	501 (12.4%)	494 (14.1%)	517 (14.7%)	626 (17.4%)	
Other Hispanic	1545 (10.5%)	460 (11.4%)	367 (10.5%)	356 (10.1%)	362 (10.0%)	
Non-Hispanic Asian	1665 (11.4%)	440 (10.9%)	411 (11.7%)	409 (11.6%)	405 (11.2%)	
Non-Hispanic Black	3174 (21.6%)	1001 (24.8%)	741 (21.1%)	711 (20.3%)	721 (20.0%)	
Non-Hispanic White	5577 (38.0%)	1476 (36.6%)	1366 (38.9%)	1387 (39.5%)	1348 (37.4%)	
Other Race	564 (3.8%)	156 (3.9%)	132 (3.8%)	131 (3.7%)	145 (4.0%)	
Education level, n (%)						< 0.001
Less than 9th grade	1261 (8.6%)	433 (10.7%)	312 (8.9%)	251 (7.1%)	265 (7.3%)	
9–11th grade	1755 (12.0%)	520 (12.9%)	422 (12.0%)	380 (10.8%)	433 (12.0%)	
High school graduate	3367 (23.0%)	986 (24.4%)	787 (22.4%)	773 (22.0%)	821 (22.8%)	
Some college or AA degree	4594 (31.3%)	1279 (31.7%)	1059 (30.2%)	1135 (32.3%)	1121 (31.1%)	
College graduate or above	3686 (25.1%)	816 (20.2%)	931 (26.5%)	972 (27.7%)	967 (26.8%)	
Smoking, n (%)						< 0.001
Yes	6268 (42.7%)	1629 (40.4%)	1404 (40.0%)	1517 (43.2%)	1718 (47.6%)	
No	8395 (57.3%)	2405 (59.6%)	2107 (60.0%)	1994 (56.8%)	1889 (52.4%)	
Hypertension, n (%)						< 0.001
Yes	5453 (37.2%)	1572 (39.0%)	1306 (37.2%)	1332 (37.9%)	1243 (34.5%)	
No	9210 (62.8%)	2462 (61.0%)	2205 (62.8%)	2179 (62.1%)	2364 (65.5%)	
Diabetes, n (%)						0.1437
Yes	2088 (14.2%)	618 (15.3%)	503 (14.3%)	480 (13.7%)	487 (13.5%)	
No	12,168 (83.0%)	3321 (82.3%)	2908 (82.8%)	2927 (83.4%)	3012 (83.5%)	
Borderline	407 (2.8%)	95 (2.4%)	100 (2.8%)	104 (3.0%)	108 (3.0%)	
Stroke, n (%)						< 0.001
Yes	570 (3.9%)	202 (5.0%)	152 (4.3%)	126 (3.6%)	90 (2.5%)	
No	14,093 (96.1%)	3886 (95.0%)	3359 (95.7%)	3385 (96.4%)	3517 (97.5%)	
Heart failure, n (%)						< 0.001
Yes	479 (3.3%)	148 (3.7%)	113 (3.2%)	159 (4.5%)	103 (2.9%)	
No	14,184 (96.7%)	3886 (96.3%)	3398 (96.8%)	3352 (95.5%)	3504 (97.1%)	
Heart attack, n (%)						< 0.001
Yes	621 (4.2%)	172 (4.3%)	141 (4.0%)	159 (4.5%)	149 (4.1%)	
No	14,042 (95.8%)	3862 (95.7%)	3370 (96.0%)	3352 (95.5%)	3458 (95.9%)	
Coronary heart disease, n (%)						< 0.001
Yes	625 (4.3%)	178 (4.4%)	161 (4.6%)	149 (4.2%)	137 (3.8%)	
No	14,038 (95.7%)	3856 (95.6%)	3350 (95.4%)	3362 (95.8%)	3470 (96.2%)	

SD, standard deviation.

### Correlation between dietary choline intake and the risk of CVD

**Figure 1:**
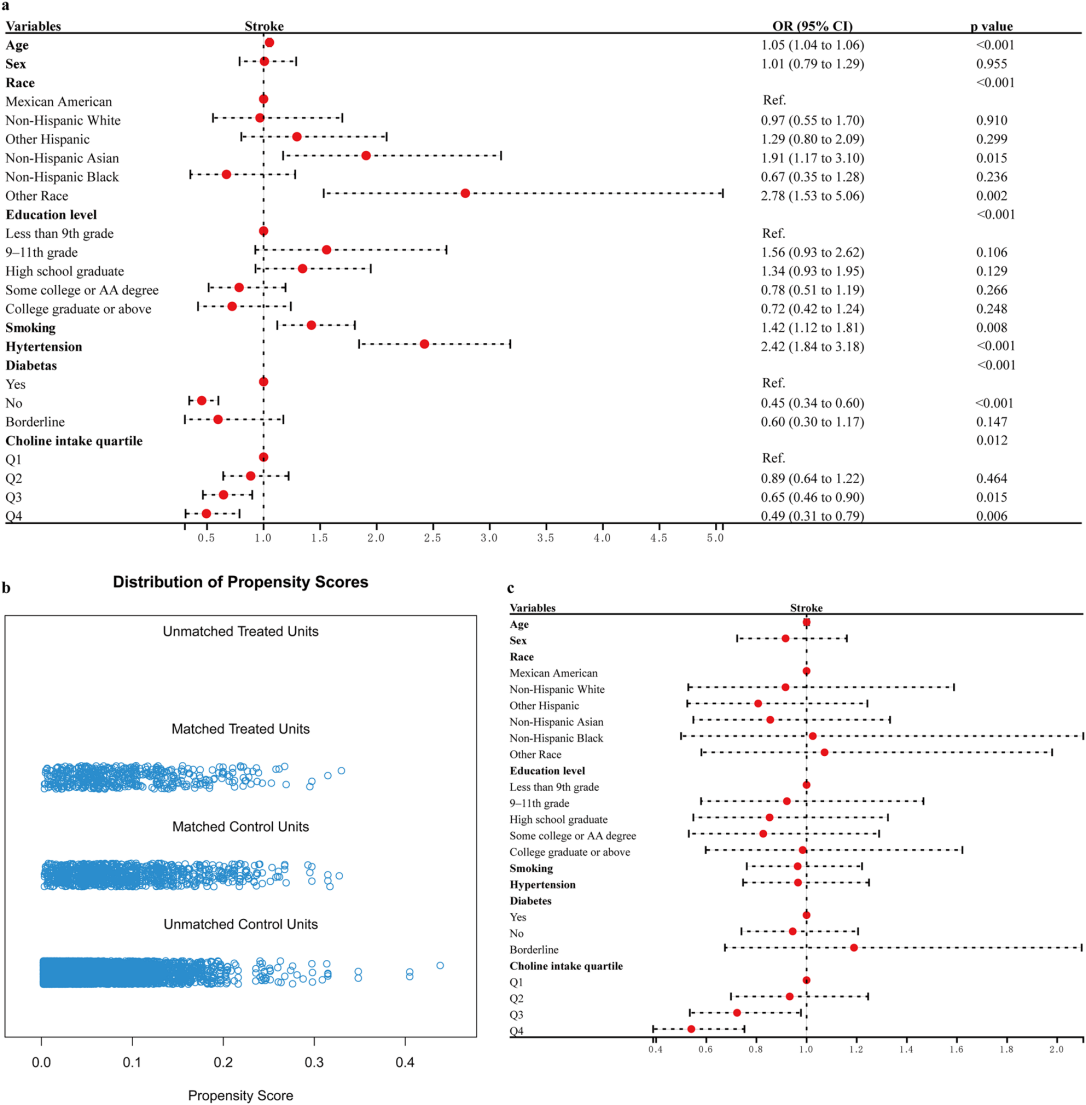
Forest plots of stroke risk factors before and after propensity score matching (PSM). (**a**) The forest plot of stroke risk factors before matching. (**b**) The distribution of propensity scores is examined for both the matched and unmatched stroke and control subjects, with a ratio of 1:2. (**c**) The forest plot of stroke risk factors after matching.

As depicted in Fig. 1a, subsequent to multivariate adjustment for all covariates outlined in Table 1, the quartile-based classification of dietary choline intake was carried out. The odds ratio (OR) for stroke in quartile 3 (303.00–413.60 mg/day) was 0.65 with a 95% confidence interval (CI) of [0.46, 0.90], while in quartile 4 (> 413.60 mg/day) it was 0.49 with a 95% CI of [0.31, 0.79] relative to quartile 1 (< 216.95 mg/day). The p value for trend across quartiles was determined to be 0.012. Apart from that, choline was only found to be associated with coronary heart disease (CHD) among the remaining three diseases in quartile 4 and the OR was 0.70 with a 95% CI of [0.51, 0.97]. The results suggested that the dietary choline intake was strongly associated with the incidence of stroke.

To further validate the association between dietary choline intake and stroke risk, we employed the propensity score matching (PSM) method to establish a comparable control group with a 1:2 ratio (Figs. 1b and S2). We included 994 and 497 participants in control group and stroke group respectively. The dietary choline intake exhibited a statistically significant difference between the control group (328 mg/day) and the stroke group (294 mg/day) with a p value less than 0.001 while no significant differences were observed for other baseline characteristics between the two groups (Table S1). The multivariate analysis results after matching showed that stroke risk of quartile 3 and 4 of dietary choline intake were 0.72 (p = 0.035) and 0.54 (p < 0.001) times that of quartile 1 (Fig. 1c).

Subsequently, a stratified approach was employed to explore potential variations in the relationship between dietary choline intake and the risk of stroke based on body composition factors, including body mass index (BMI) and waist circumstance (WC). BMI and WC exhibited significant effect modifications on the relationship between choline intake and stroke (Tables 2 and S2). Notably, a significant association between choline intake and stroke was observed exclusively in individuals classified as relatively obese (BMI ≥ 25 kg/m^2^ and WC ≥ 102 cm).

**Table 2:** Subgroup analysis between dietary choline intake and the risk of stroke stratified by BMI.

	BMI < 25	BMI ≥ 25, < 30	BMI ≥ 30
Choline intake quartile			
p value	0.940	0.043	0.006
Quartile1	Ref.	Ref.	Ref.
Quartile2	1.05 (0.55, 1.99)	0.76 (0.45, 1.29)	0.96 (0.62, 1.49)
p value	0.892	0.308	0.8695
Quartile3	1.01 (0.54, 1.89)	0.52 (0.29, 0.93)	0.73 (0.47, 1.14)
p value	0.985	0.028	0.1652
Quartile4	1.24 (0.60, 2.56)	0.47 (0.26, 0.85)	0.44 (0.27, 0.73)
p value	0.557	0.013	0.0015

CI, confidence interval; OR, odds ratio; BMI, body mass index; Ref., reference.

### Nonlinear association between dietary choline intake and the risk of stroke

**Figure 2:**
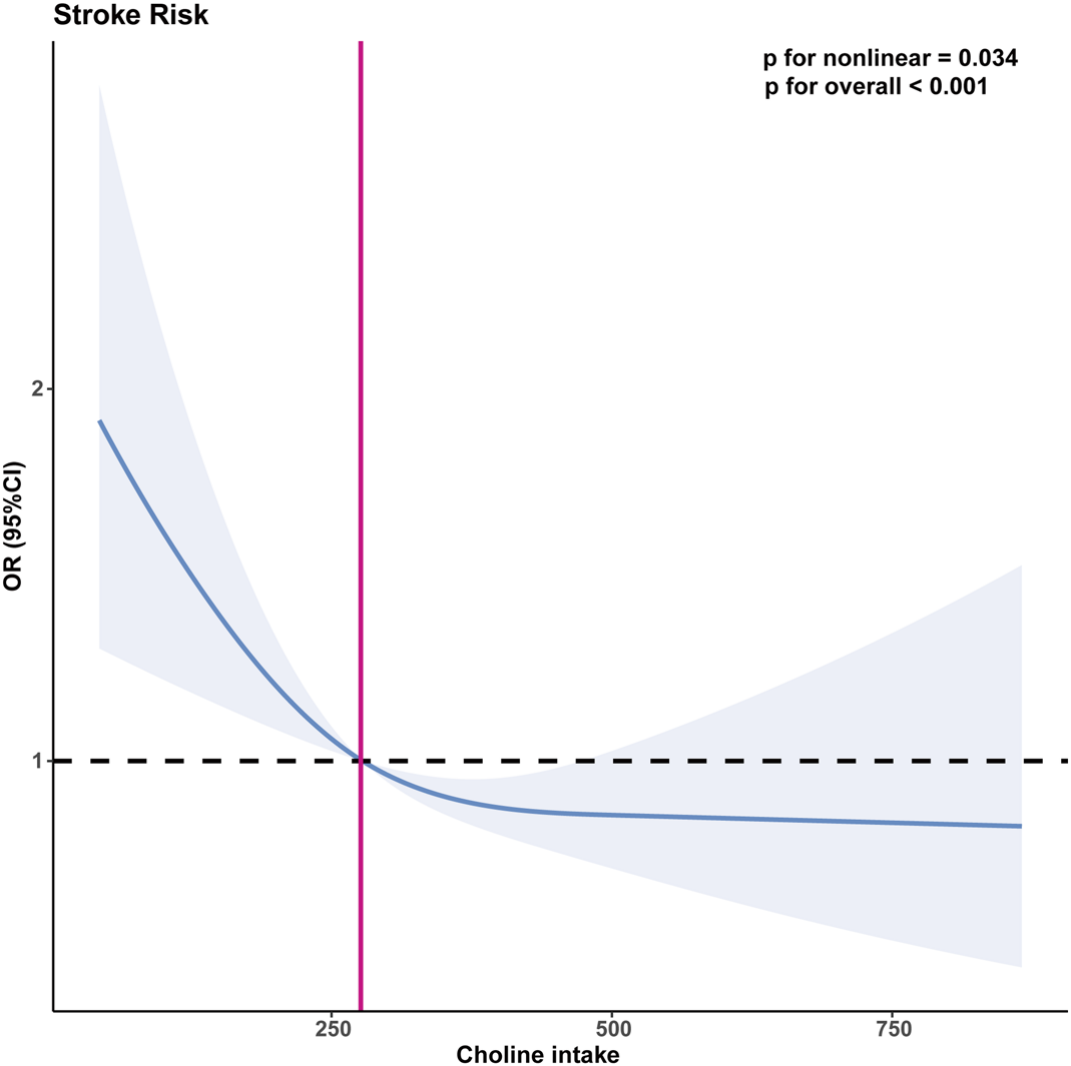
Restricted cubic spline (RCS) models adjusting for sex, age, race, education, smoking, hypertension, and diabetes.. The 95% confidence intervals of the adjusted odds ratios are visually depicted by the light purple-shaded region. including.

To better elucidate the relationship between dietary choline intake and the risk of stroke, we employed the restricted cubic spline (RCS) analysis following the PSM. We observed a non-linear negative correlation between the prevalence of stroke and the increase in dietary choline intake (p for non-linearity = 0.034), as illustrated in Fig. 2 using a spline smoothing plot. The adjusted model revealed an L-shaped association between dietary choline intake and stroke risk.276 mg/day of dietary intake was sufficient to reduce the risk of stroke.

### Mendelian randomization revealed the causal effect of choline metabolites on body composition

**Figure 3:**
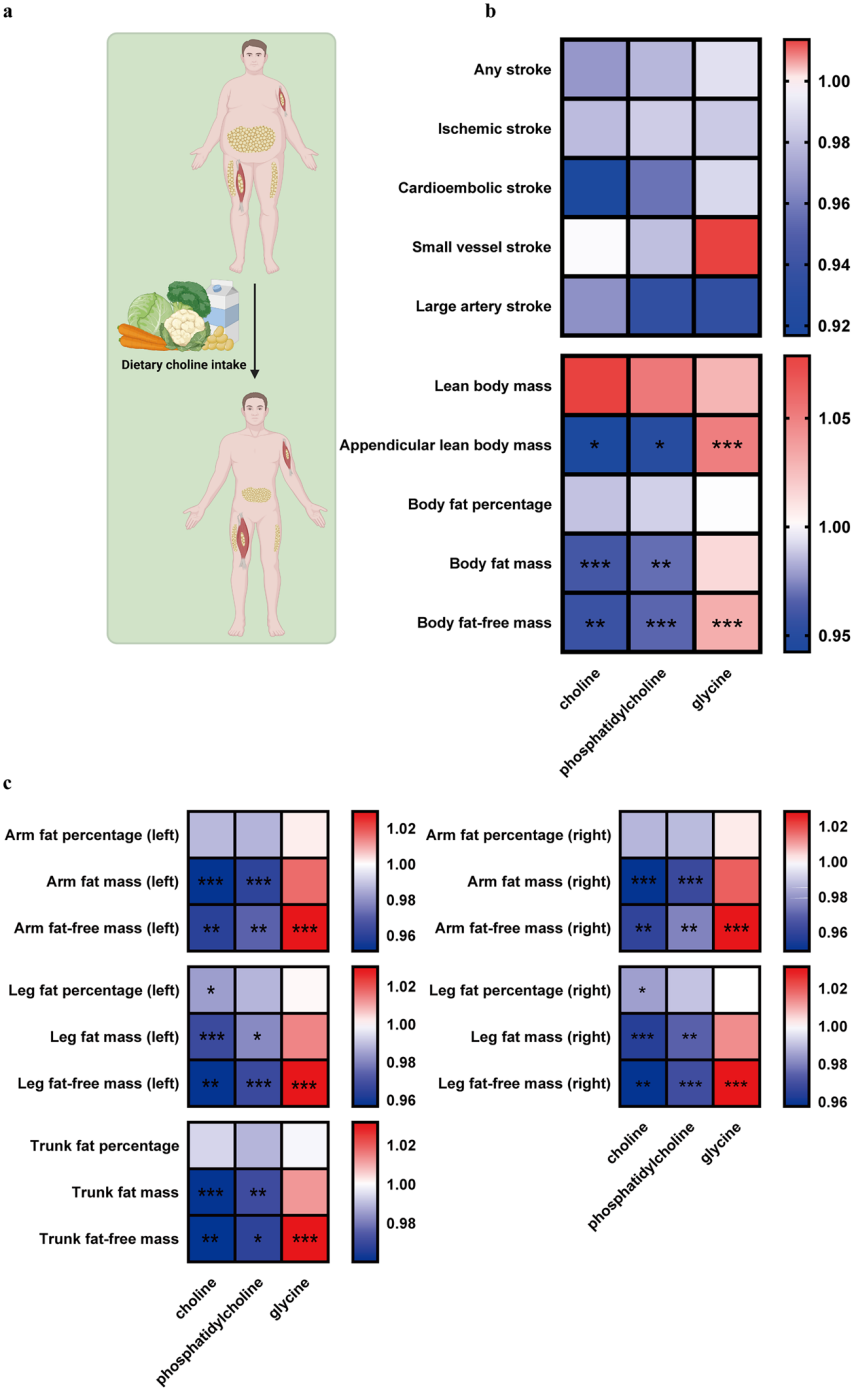
Causal relationship between genetically predicted choline metabolites and the risk of stroke and the body composition. (**a**) A schematic representation illustrating the relationship between choline metabolites and body composition. (**b**) Causal effects of choline metabolites on stroke and body composition. The effects are exhibited in heatmap with odds ratio. Choline metabolites encompass choline, phosphatidylcholine, and glycine. Stroke is classified into five distinct types, namely any stroke, ischemic stroke, cardioembolic stroke, small vessel stroke, and large artery stroke. Body composition encompasses lean body mass, appendicular lean body mass, body fat percentage, body fat mass, and body fat-free mass. (**c**) Causal effects of choline metabolites on body fat composition. The effects are exhibited in heatmap with odds ratio. Choline metabolites encompass choline, phosphatidylcholine, and glycine. Body fat composition encompasses fat percentage, mass, and fat-free mass of right/left arm/leg and trunk.

The aforementioned results, which indicated that the effect of choline on stroke differs in different groups categorized based on BMI and WC, suggested that the protective effect of choline on stroke may be mediated by alterations in body composition (Fig. 3a). Hence, we explored the association between dietary choline intake and several body composition indicators exhibited in Fig. S3 and the results showed that all indicators were significant, expect for BMI. To validate the causal effect of choline on stroke and body composition, a series of two-sample mendelian randomization (MR) analysis were conducted using three choline metabolites, including total choline, phosphatidylcholine, and glycine. We found that no causal relationship between choline metabolites and stroke but identified the causal effect on appendicular lean body mass, body fat mass and body fat-free mass. Total choline and phosphatidylcholine exhibited negative effects on appendicular lean body mass, body fat mass and body fat-free mass while opposite effects were observed in glycine (Fig. 3b). Furthermore, the similar causal effects between choline metabolites and fat percentage, fat mass and fat-free mass of arms, legs and trunk were observed (Fig. 3c). These results suggested the protective effect of choline on stroke may be mediated by alterations of the fat mass and distribution.

### Choline metabolism pathway was involved in the immune response and neural regeneration following stroke

**Figure 4:**
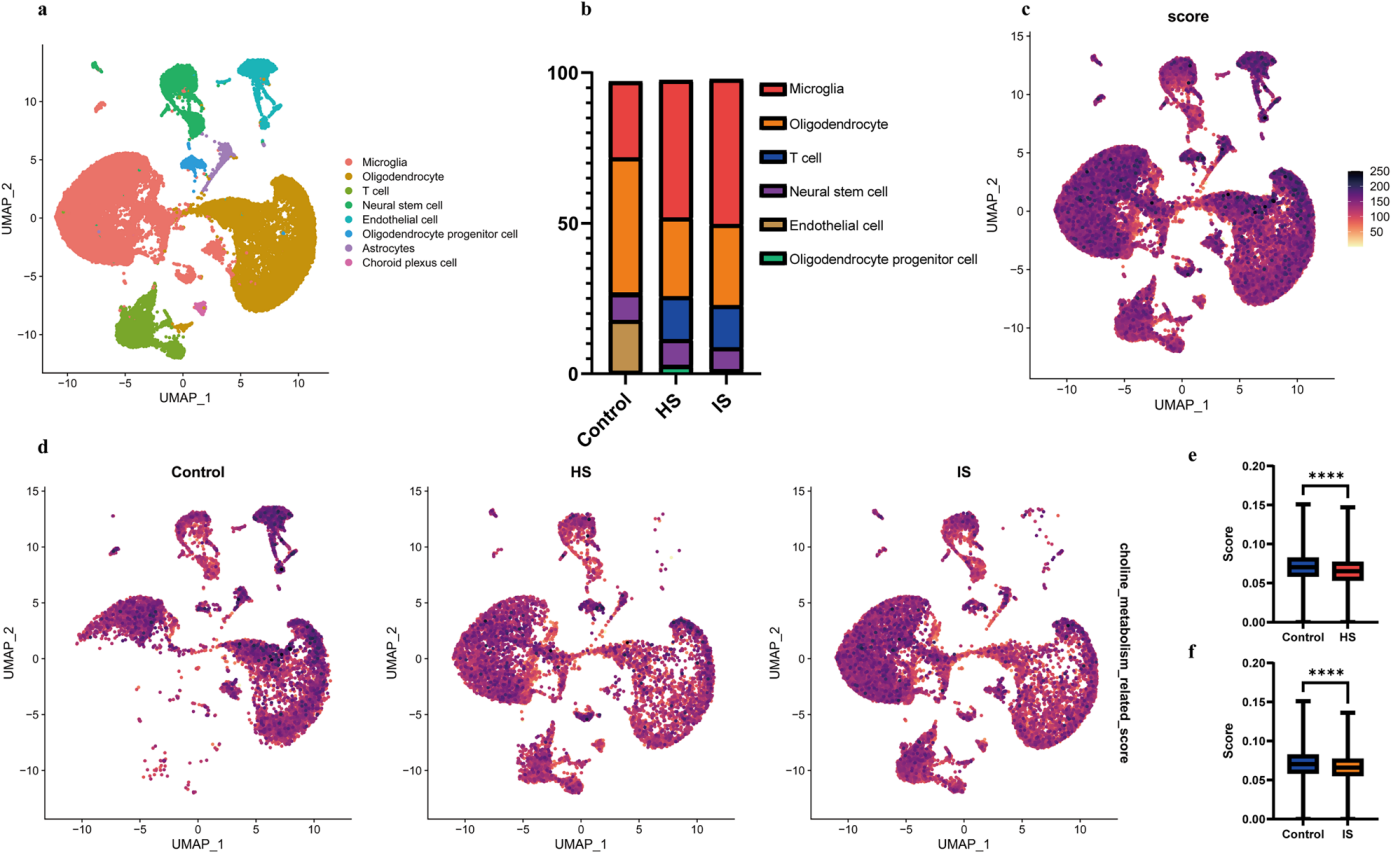
Single-cell transcriptomic analysis and the assessment of choline pathway score in post-stroke brain samples. (**a**) The uniform manifold approximation and projection (UMAP) plot of the identified cell clusters in brain tissue from healthy control, ischemic stroke, and hemorrhagic stroke groups. (**b**) Relative proportion of each cell cluster of healthy control group, ischemic stroke group, and hemorrhagic stroke group. (**c**) UCell scores of choline metabolism gene signature of single cell from the total of healthy control group, ischemic stroke group, and hemorrhagic stroke group. (**d**) UCell scores of choline metabolism gene signature of single cell from healthy control (Control) group, ischemic stroke (IS) group, and hemorrhagic stroke (HS) group, respectively. (**e**) Bar plot showing the choline metabolism gene signature UCell scores of control group and ischemic stroke group. (**f**) Bar plot showing the choline metabolism gene signature UCell scores of control group and hemorrhagic stroke group. p values were determined by a two-tailed Student's t-test. *, p < 0.05; **, p < 0.01; ***, p < 0.001 ****, p < 0.0001.

Several studies have shown that a higher plasma choline level is correlated with a reduced risk of post-stroke cognitive impairment^18^ and depression^32^. These results suggested a potential association between choline metabolism pathway and stroke prognosis, however, underlying mechanisms remains unclear. Here, we identified a signature of choline metabolism genes from KEGG, AmiGO2, and Reactome Pathway databases (Table S3) and obtained the single cell RNA-sequencing data consisting of healthy mice, hemorrhagic stroke (HS) and ischemic stroke (IS) models. As shown in Fig. 4a, a total of 8 main cell clusters were finally identified. The proportion of microglia and T cells obviously increased in both HS and IS groups (Fig. 4b), indicating that the immune response and inflammatory processes play crucial roles after stroke. To evaluate choline metabolism-related gene signature in single-cell dataset, UCell and AUCell were employed (Figs. 4c and S4a). We observed the choline metabolism-related gene signature was mostly enriched in microglia, T cell and endothelial cell (Figs. 4d and S4b) and the scores of both HS and IS groups were significantly decreased compared to the Control group (Figs. 4e, f and S4c, d). We then analyzed the differentially expressed genes (DEGs) of endothelial cells and the Molecular Complex Detection (MCODE) algorithm was employed to identify highly interconnected modules (Fig. S5a). The gene ontology (GO) enrichment (Fig. S5b) of the choline metabolism-related DEGs involved green module (Fig. S5a) showed that choline metabolism pathway was associated with angiogenesis. Besides, the results of MCODE analysis (Fig. S5c) and GO enrichment (Fig. S5d) of choline metabolism-related DEGs from microglia suggested that choline metabolism pathway can regulate the immune response, which was found to play a double-edged sword role^33^. Moreover, T cells were crucial in the pathophysiological processes of stroke^34^. The MCODE analysis (Fig. S5e) and GO enrichment (Fig. S5f–h) of choline metabolism-related DEGs in T cells indicated that choline metabolism pathway plays a vital role in the regulation of both immune response and angiogenesis, which is consistent with previous findings^34,35^.

### Choline reduced CVD risk by modulating adipose tissue functionality

**Figure 5:**
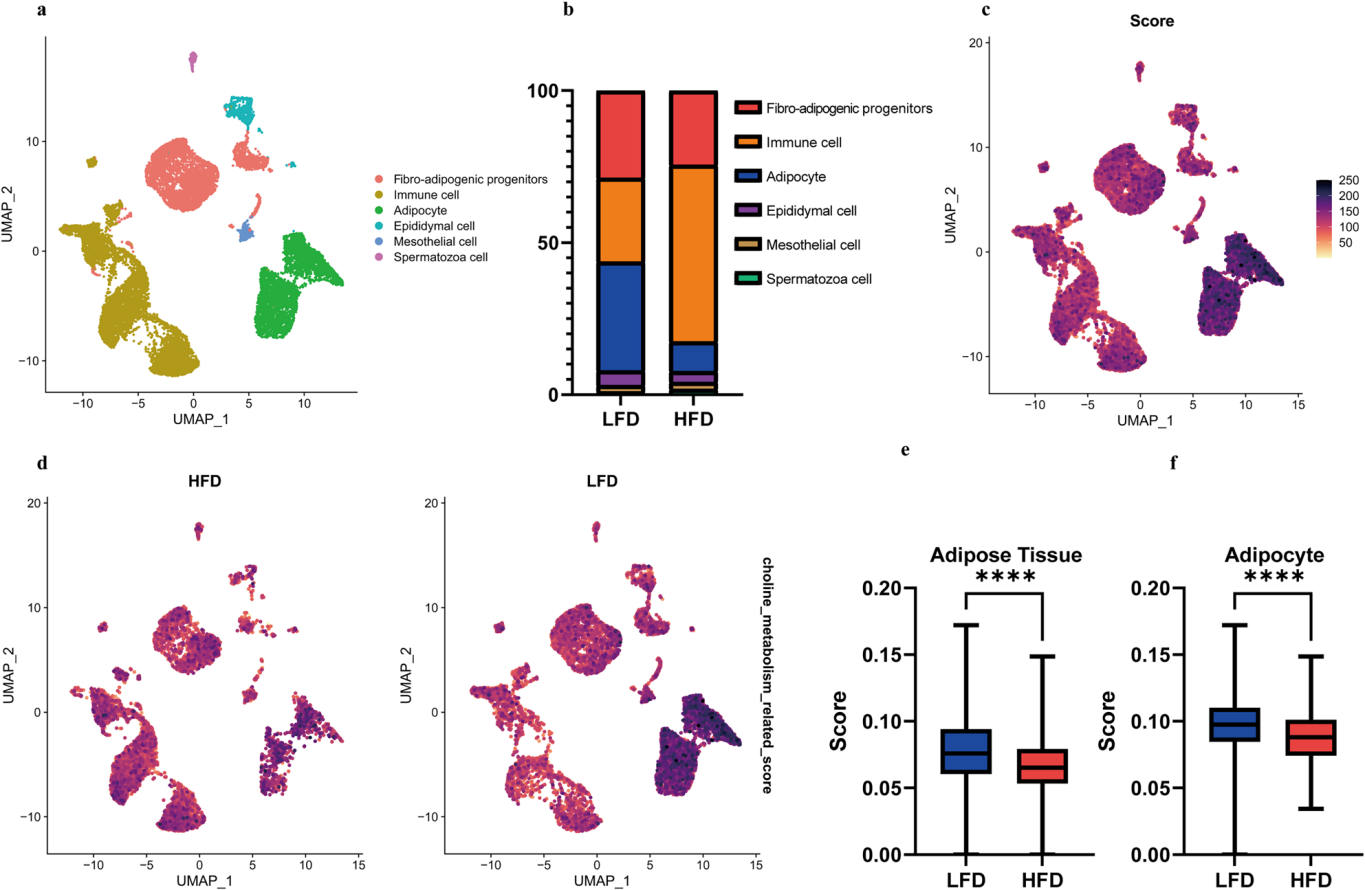
Single-cell transcriptomic analysis and the assessment of choline pathway score in adipose tissue from high-fat diet (HFD) fed mice and low-fat diet (LFD) fed mice. (**a**) The uniform manifold approximation and projection (UMAP) plot of the identified cell clusters in adipose tissues from high-fat diet fed group and low-fat diet fed group. (**b**) Relative proportion of each cell cluster of high-fat diet fed group and low-fat diet fed group. (**c**) UCell scores of choline metabolism gene signature of single cell from both high-fat diet fed group and low-fat diet fed group. (**d**) UCell scores of choline metabolism gene signature of single cell from high-fat diet fed group and low-fat diet fed group, respectively. (**e**) Bar plot showing the choline metabolism gene signature UCell scores of adipose tissues from high-fat diet fed group and low-fat diet fed group. (**f**) Bar plot showing the choline metabolism gene signature UCell scores of adipocytes from high-fat diet fed group and low-fat diet fed group. p values were determined by a two-tailed Student's t-test. *, p < 0.05; **, p < 0.01; ***, p < 0.001 ****, p < 0.0001.

**Figure 6:**
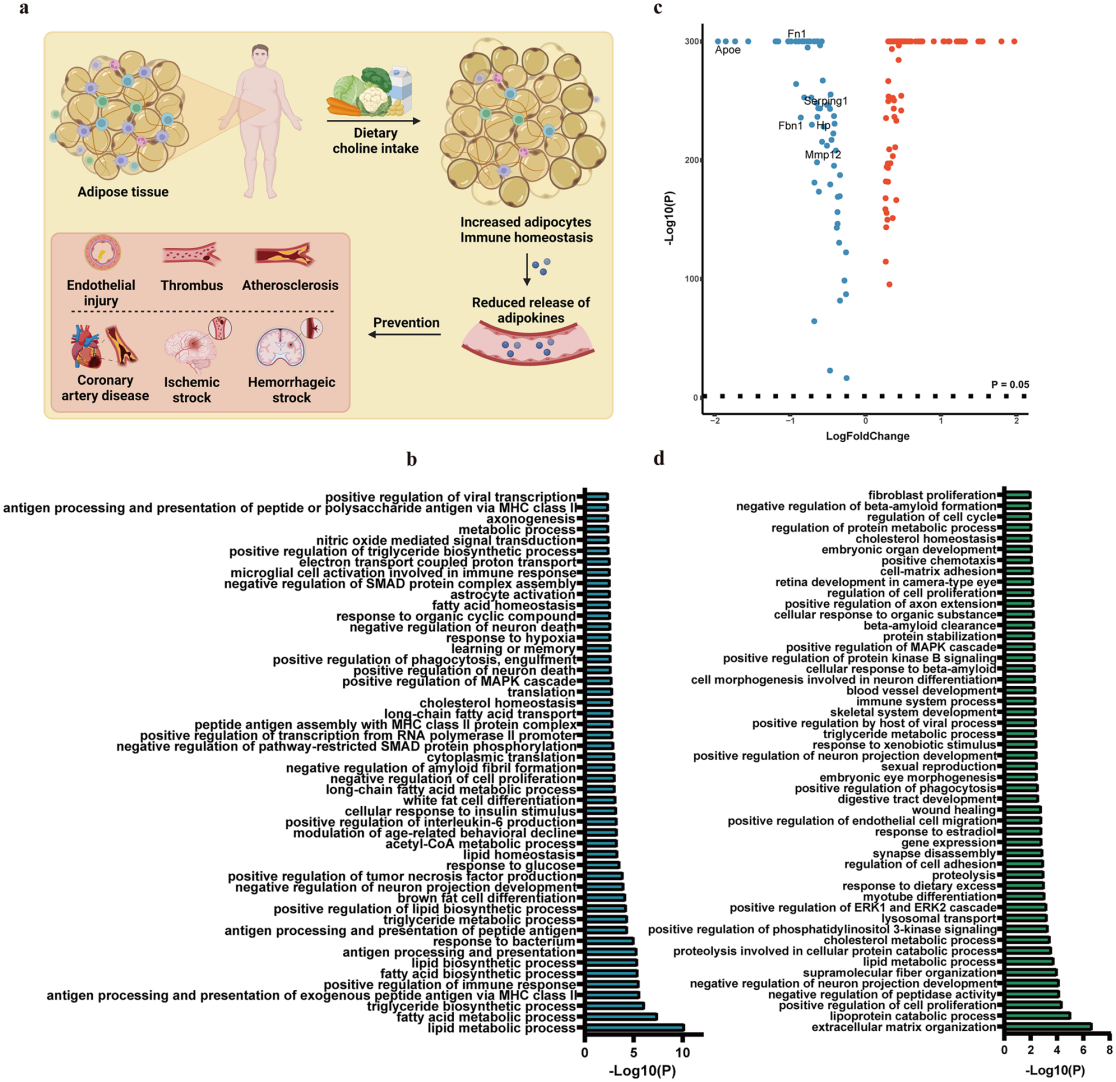
Identification of adipocyte functionality under high-fat diet (HFD) and low-fat diet (LFD) conditions. (**a**) A schematic representation illustrating the relationship between choline, functionality of adipocyte, and cardiovascular disease incidence. (**b**) Gene ontology (GO) enrichment of differentially expressed genes in adipocyte between low-fat diet fed group and high-fat diet fed group. (**c**) Volcano plot showing differentially expressed genes encoding secreted proteins in adipocyte compared to other cell clusters. (**d**) Gene ontology enrichment of differentially expressed genes encoding secreted proteins in adipocyte compared to other cell clusters.

Our results showed that dietary choline intake can significantly reduce the risk of stroke in populations with higher BMI or WC, hence, we aimed to unravel the underlying mechanisms here. We acquired the single cell RNA-sequencing data of adipose tissue from high-fat diet (HFD) fed mice and low-fat diet (LFD) fed mice. A total of 6 distinct cell clusters were identified (Fig. 5a), revealing noticeable differences in the proportions of immune cells and adipocytes between the two groups (Fig. 5b). These findings strongly suggested that the HFD significantly alters the immune microenvironment in adipose tissue. The results from UCell (Fig. 5c) and AUCell (Fig. S6a) analysis indicated that the choline metabolism-related gene signature was primarily enriched in the adipocyte cell cluster (Figs. 5d and S6b), and that the score was reduced in both adipose tissue and adipocytes under HFD conditions (Figs. 5e, f and S6c, d). Based on the result, adipocytes appeared to be the primary cell type regulated by the choline metabolism pathway, which played a positive role in body composition modification and CVD prevention. To systematically analyze and interpret functional pathways and biological processes associated with adipocyte, GO enrichment analysis was employed on DEGs of adipocyte and the results were mainly associated with glucose/lipid metabolism-related pathways, and immune response (Fig. S7a). The MCODE analysis of DEGs in adipocyte revealed the involvement of choline metabolism-related genes (Fig. S7b), and subsequent GO enrichment analysis of the corresponding module demonstrated their primary roles in pathways related to immune response, glucose/lipid metabolism-related pathways, insulin-related pathways, and endothelial migration (Fig. S7c). These findings highlighted the potential link between the choline metabolism pathway and several adipose tissue characteristics, such as mass, area, distribution, immune microenvironment, as well as the adipose tissue mediated susceptibility to CVD (Fig. 6a). Then we analyzed the DEGs of adipocyte between HFD group and LFD group and performed GO enrichment. These results revealed that these genes primarily participate in glucose/lipid metabolism-related pathways, adipose tissue differentiation, and immune response (Fig. 6b). This suggested a significant alteration in the biological function of adipocytes under HFD conditions, which may contribute to changes in biological characteristics of adipose tissue and increase the risk of CVD. Adipose tissue harbors the capability to biosynthesize and release a diverse array of hormones, cytokines, extracellular matrix proteins, and growth and vasoactive factors known as adipokines. These adipokines play a crucial role in regulating various physiological and pathophysiological processes that are intricately associated with CVD^36,37^. Here, we conduct a analysis of DEGs encoding secreted proteins in the adipocyte, among which several have been validated to be associated with increased CVD risk, including apoE, fibronectin, serpinG1, fibrillin-1, haptoglobin, and MMP12^38–44^ decreased (Fig. 6c). The GO enrichment analysis of DEGs encoding secreted proteins revealed that these proteins primarily contribute to the maintenance of nervous system homeostasis, blood vessel development, wound healing, as well as glucose/lipid metabolism-related pathways (Fig. 6d), suggesting the choline metabolism pathway may modulate the functionality of adipocyte to reduce the risk of CVD.

## Discussion

The inclusion of choline in one's diet has been previously hypothesized as a potential risk factor for CVD because of its metabolic conversion by gut microbiota and the liver into TMAO^12,13^. Additionally, a clinical study has shown that phosphatidylcholine can be metabolized by the gut microbiota into TMAO, which has been associated with an elevated risk of CVD incidence^45^. Current studies suggest that individuals who are susceptible to CVD should avoid the consumption of choline and phosphatidylcholine-rich foods, such as meat and egg yolks^46^. However, insufficient choline stores can lead to a decreased capacity to methylate homocysteine into methionine, resulting in elevated plasma levels of homocysteine^47^. Elevated levels of homocysteine have been observed to be linked to a heightened vulnerability to CVD, specifically stroke^48–50^. The ingestion of choline, whether through dietary sources or supplements, has been linked to a decrease in homocysteine levels. This association holds true regardless of whether choline is consumed alone or in combination with other nutrients^51^. The current recommended AI for daily dietary choline was primarily based on its muscle- and liver-protective effect. Consequently, the optimal dietary intake quantity of choline for reducing the risk of CVD and improving prognosis remains unresolved and requires further investigation. In this study, our result of the RCS analysis indicated a L-shaped dose–response effect, with more than 276 mg/day being sufficient to reduce the risk of stroke.

Given that the protective effect of choline was predominantly observed in individuals with higher BMI and WC, it is hypothesized that this protective effect may be mediated indirectly through the improvement of body composition, specifically by reducing BMI, WC, fat mass index (FMI), and increasing lean mass index (LMI). Our analysis unveiled a significant correlation between the intake of dietary choline and various measures of body composition. These measures encompassed fat mass index (FMI), lean mass index (LMI), waist circumference (WC), as well as the mass, area, and volume of total abdominal, visceral, and subcutaneous fat. Previous studies have also reported similar findings^52–54^, and it is likely that the observed effect is associated with improved lipid metabolism^55^. To delve deeper into the causal impact of choline and its associated metabolites on stroke and body composition, we conducted MR analysis. Our results indicated that genetically predicted levels of choline and its metabolites, such as phosphatidylcholine and glycine, were found to be correlated with modified body composition. However, no significant association was observed between these factors and the occurrence of stroke. To the best of our knowledge, this study presented the initial comprehensive examination of the correlation and causality between choline and the risk of stroke, as well as its impact on body composition. Furthermore, this study is the first to investigate the potential mediation effect of body composition on the correlation.

To gain a deeper understanding of the impact of the choline metabolism pathway on the biological characteristics of adipose tissue and its role in refining body composition to prevent stroke, we conducted an analysis of adipose tissue samples obtained from mice fed a high-fat diet and mice fed a low-fat diet. The results of this study provided evidence that the adipocyte served as the principal target responding to choline metabolism pathway. Additionally, it has been noted that choline metabolism pathway possessed the capacity to modulate multiple pathways linked to glucose and lipid metabolism, insulin, and immune response. These pathways have been recognized as having a significant impact on systemic metabolism and body composition. Besides, instead of focusing solely on the overall quantity of adipose tissue, it is more pertinent to consider the quality and functionality of adipose tissue when assessing the risk of CVD^56–58^. An analysis was conducted to investigate the changes in adipocyte functionality between the groups exposed to a HFD and an LFD. Our research findings indicated that the choline metabolism pathway was disrupted in the group subjected to an HFD, resulting in compromised hypoxia response, insulin response, fatty acid, glucose, lipid metabolism homeostasis, and regulation of fat differentiation. In previous studies, it has been well-documented that proteins released by adipose tissue have a substantial impact on systemic metabolism and the pathogenesis of various CVD. Our findings were consistent with previous studies, as we have observed decreased levels of apoE, fibronectin, serpinG1, fibrillin-1, haptoglobin, and MMP12 in adipocytes compared to other cell clusters. These proteins have previously been associated with several CVD and related pathological mechanisms, including endothelial injury and atherosclerosis^38–44^. These findings offered empirical evidence to support the hypothesis that the intake of choline may exert a dual impact on body composition, while also improving the functionality of adipose tissue. Consequently, this may have potential implications for the prevention of CVD.

Choline has been acknowledged as a neuroprotective nutrient with the capacity to improve cognitive function in infants and safeguard adults against neuropathological changes associated with Alzheimer's disease. Additionally, it has been found to mitigate neurological impairments related to epilepsy, fetal alcohol syndrome, and genetic disorders such as Down and Rett syndromes^59^, suggesting that choline may play a role in regulating the interaction between various brain cells following a experience of stroke, thereby promoting tissue repair and regeneration. We conducted an analysis of cellular changes in the brain of both healthy control and post-stroke models, specifically focusing on ischemic stroke and hemorrhagic stroke. The choline metabolism pathway exhibited significant enrichment in various cell types within the brain, including endothelial cells, microglia, and T cells. Following the occurrence of a stroke, there was a discernible change in the distribution of these three cellular clusters, indicating their potential significance in the response to choline. Further analysis of the protein–protein networks have elucidated the involvement of the choline metabolism pathway in various biological processes. Specifically, it has been found to play a role in blood vessel morphogenesis and development in endothelial cells, regulation of adaptive immune response in microglia, and inflammatory regulation and endothelial proliferation in T cells. These findings suggested that the intake of choline may have a significant impact on maintaining the immune microenvironment homeostasis in the brain following stroke. Besides, it may also contribute to the stimulation of angiogenesis, leading to the formation of new blood vessels around the site of injury and ultimately improving neurogenesis and improved neurological function.

However, it is imperative to acknowledge the limitations of our research. The study’s assessment of dietary choline intake was based on two 24-h recalls, a methodology that has the potential to introduce recall bias and may not provide an accurate representation of an individual's habitual choline consumption. In addition, the choline content in food can fluctuate based on numerous factors, which may yield inaccurate outcomes. The study exclusively concentrated on the analysis of choline intake and did not investigate any possible association between dietary patterns and the occurrence of stroke incidents. Numerous factors, including dietary fiber and fat, have the potential to exert a substantial influence on the onset and advancement of various stroke subtypes. Further investigations will be undertaken to explore the correlation between diet pattern and various subtypes of stroke, given the limited current data. Despite the inclusion of multiple covariates in the analysis model, it was not feasible to completely mitigate the confounding effects of unaccounted or unidentified factors.

In the present study, we have acquired innovative perspectives indicating that the consumption of choline-rich foods plays a pivotal role in reducing the likelihood of stroke occurrence. The discrepancy can be explained by the dietary choices regarding choline consumption. Choline is primarily present in meat, and it is important to acknowledge that excessive meat consumption has been strongly linked to an elevated susceptibility to a range of diseases, including cancer and CVD^60–62^. To optimize choline intake while mitigating the potential drawbacks of excessive meat consumption, it is recommended to include plant-based sources such as cruciferous vegetables, soybeans, and nuts in one's dietary regimen. Future investigation should focus on the refinement of a diet to ensure sufficient choline intake and to improve choline metabolism, for example by regulating the microbiota to produce less TMAO. This may represent a new strategy for the prevention and treatment of stroke.

### Supplementary Information

Supplementary Information.

## Data Availability

The datasets generated and analyzed in the current study are available at NHANES website: https://www.cdc.gov/nchs/nhanes/index.htm. We used the IEU OpenGWAS at website: https://gwas.mrcieu.ac.uk/. Single cell RNA-sequencing datasets were obtained from Gene Expression Omnibus (GEO) database under the accession number GSE167593 and GSE160729.
