# Supplementary figures and images for: Choline consumption reduces CVD risk via body composition modification
Source: Sci Rep. 2024 Jul 12;14:16152. doi: 10.1038/s41598-024-66039-4 (PMC11245612; doi:10.1038/s41598-024-66039-4)

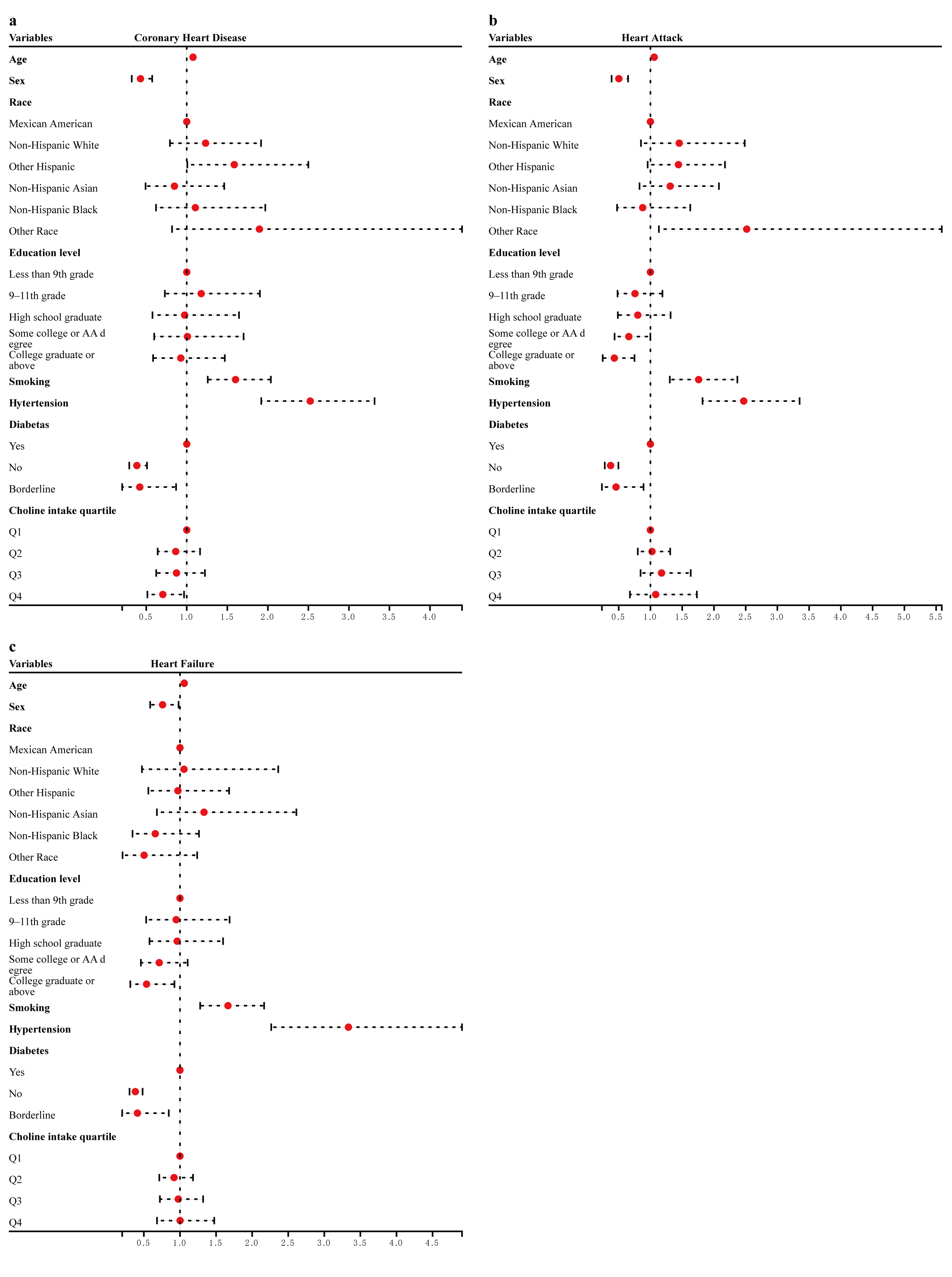

Supplement: Supplementary file 1 — Supplementary Information. [file 41598_2024_66039_MOESM1_ESM.zip › Figure S1.tif]

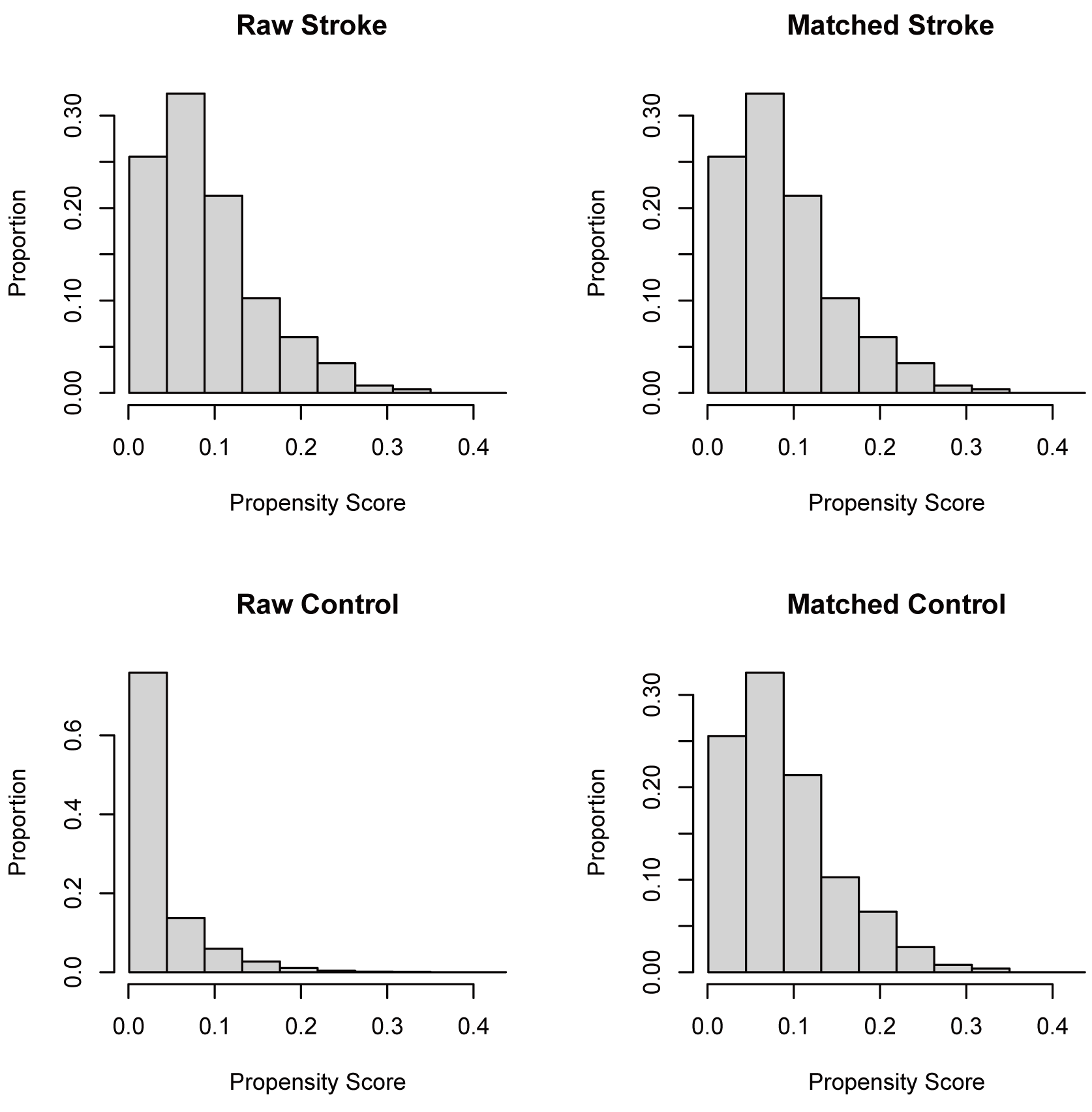

Supplement: Supplementary file 1 — Supplementary Information. [file 41598_2024_66039_MOESM1_ESM.zip › Figure S2.tif]

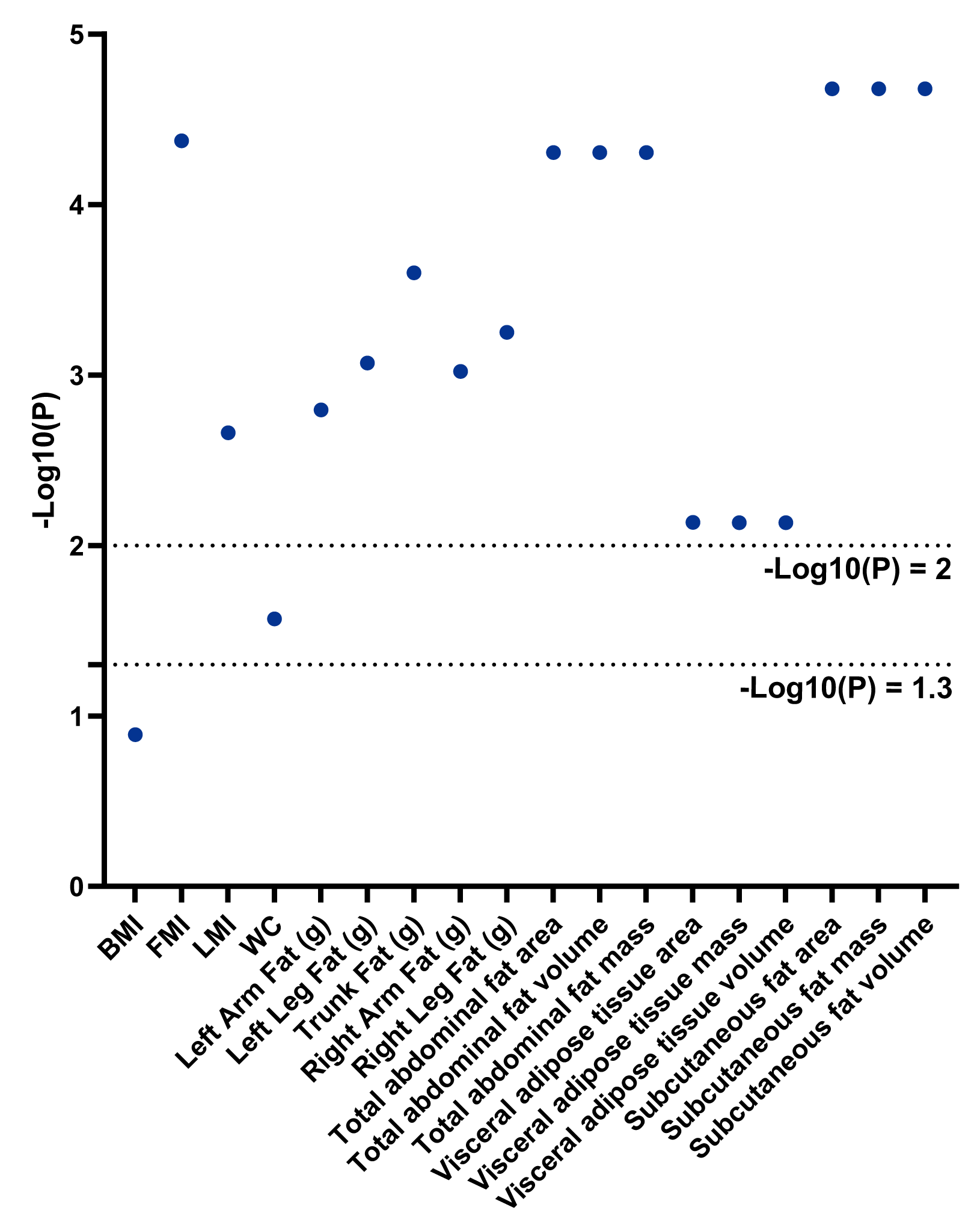

Supplement: Supplementary file 1 — Supplementary Information. [file 41598_2024_66039_MOESM1_ESM.zip › Figure S3.tif]

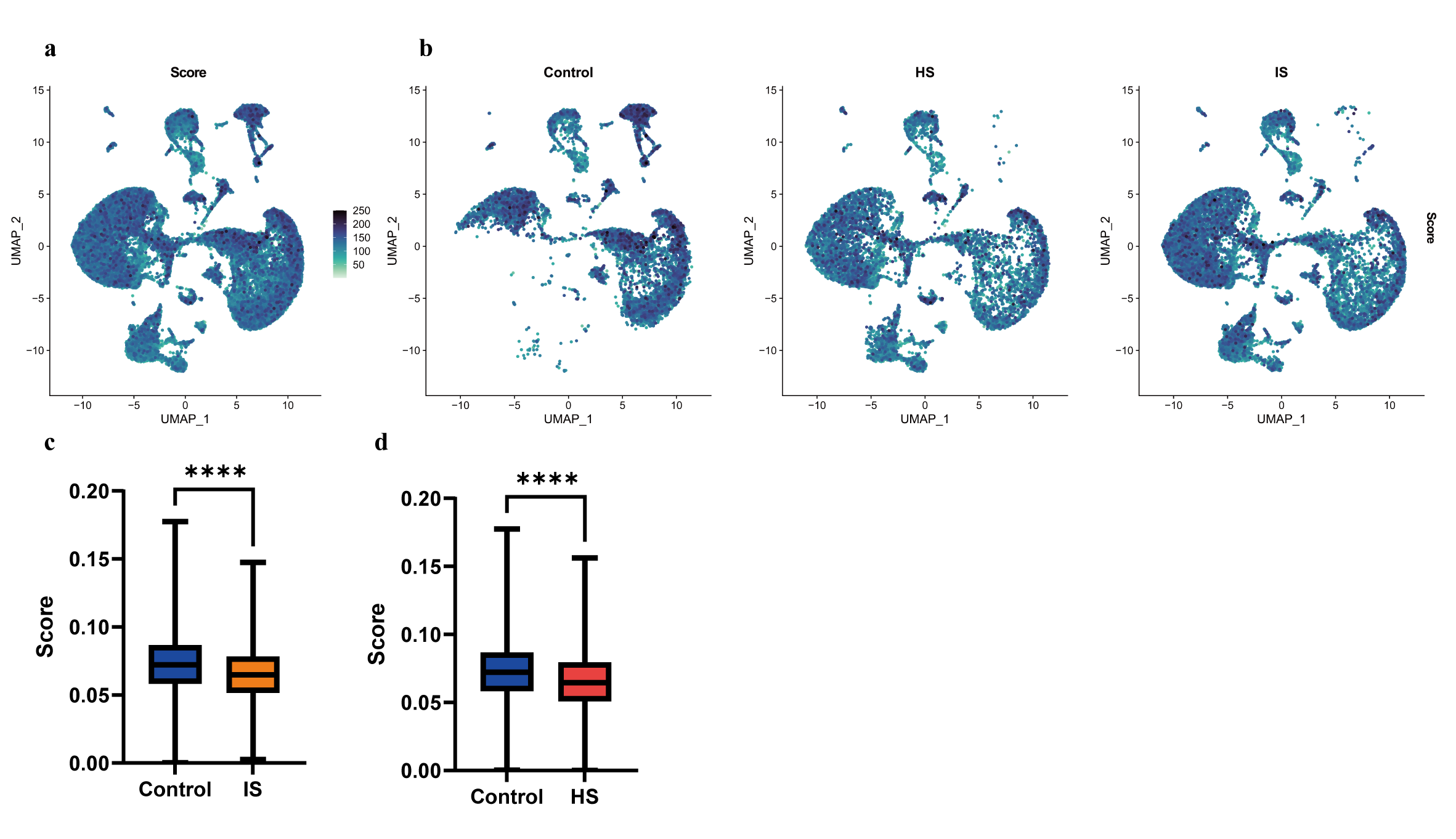

Supplement: Supplementary file 1 — Supplementary Information. [file 41598_2024_66039_MOESM1_ESM.zip › Figure S4.tif]

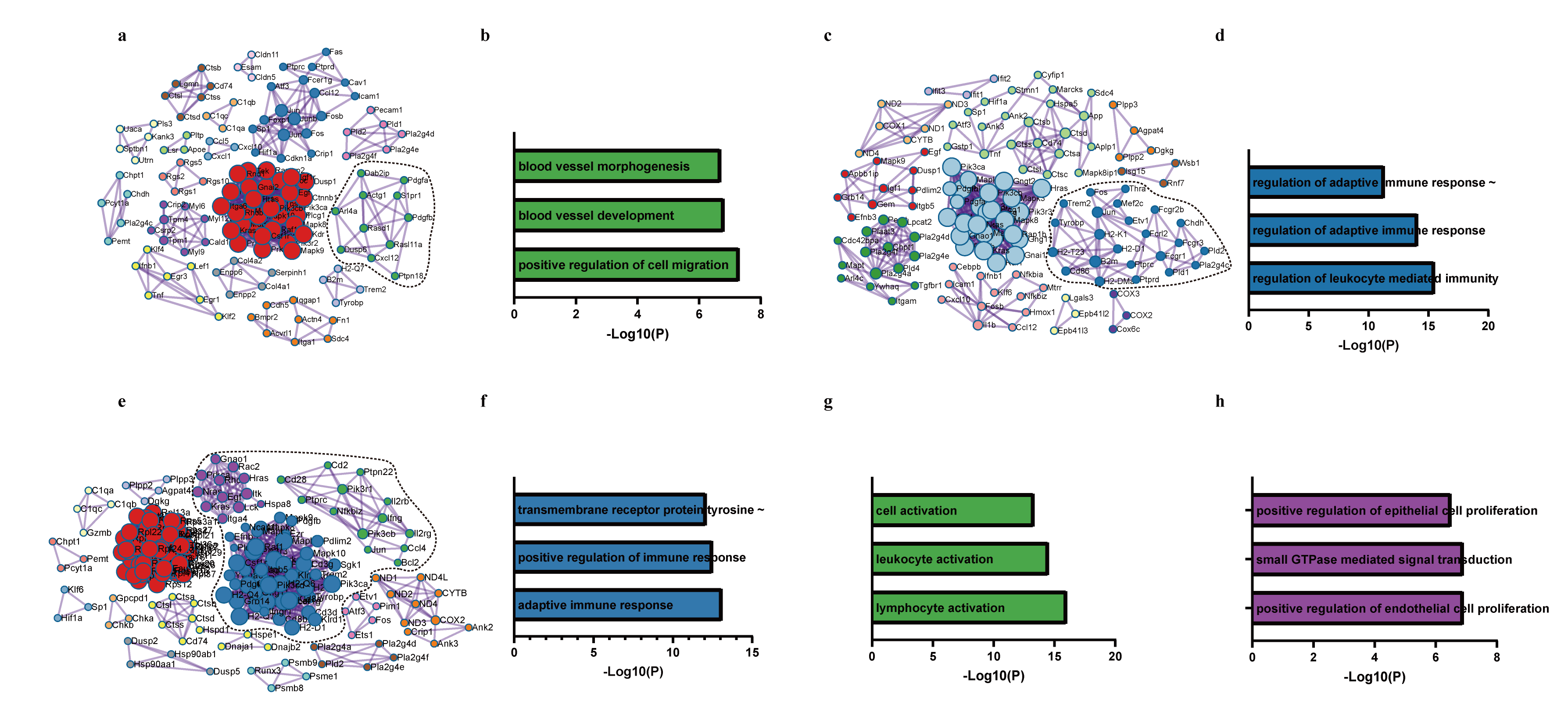

Supplement: Supplementary file 1 — Supplementary Information. [file 41598_2024_66039_MOESM1_ESM.zip › Figure S5.tif]

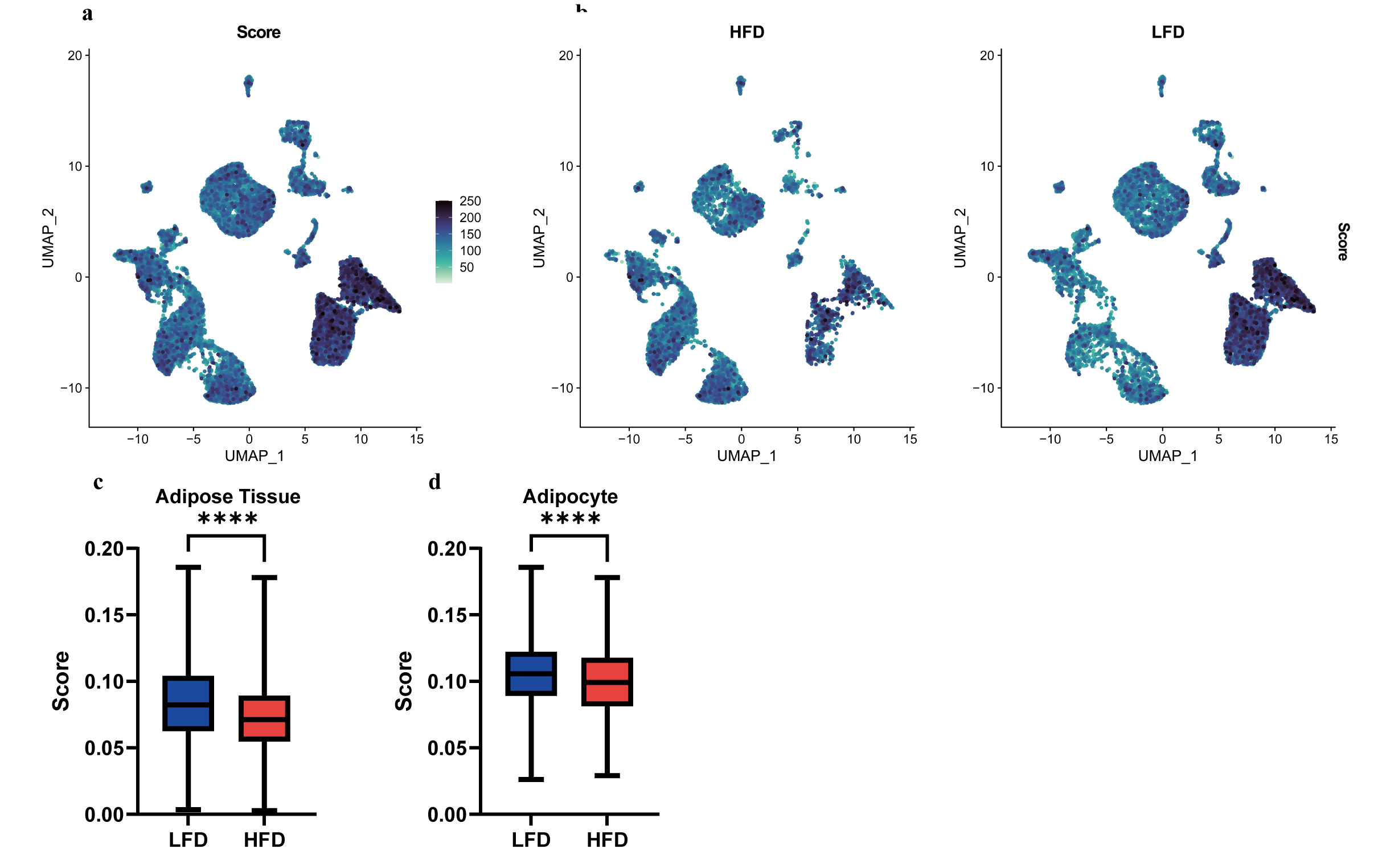

Supplement: Supplementary file 1 — Supplementary Information. [file 41598_2024_66039_MOESM1_ESM.zip › Figure S6.tif]

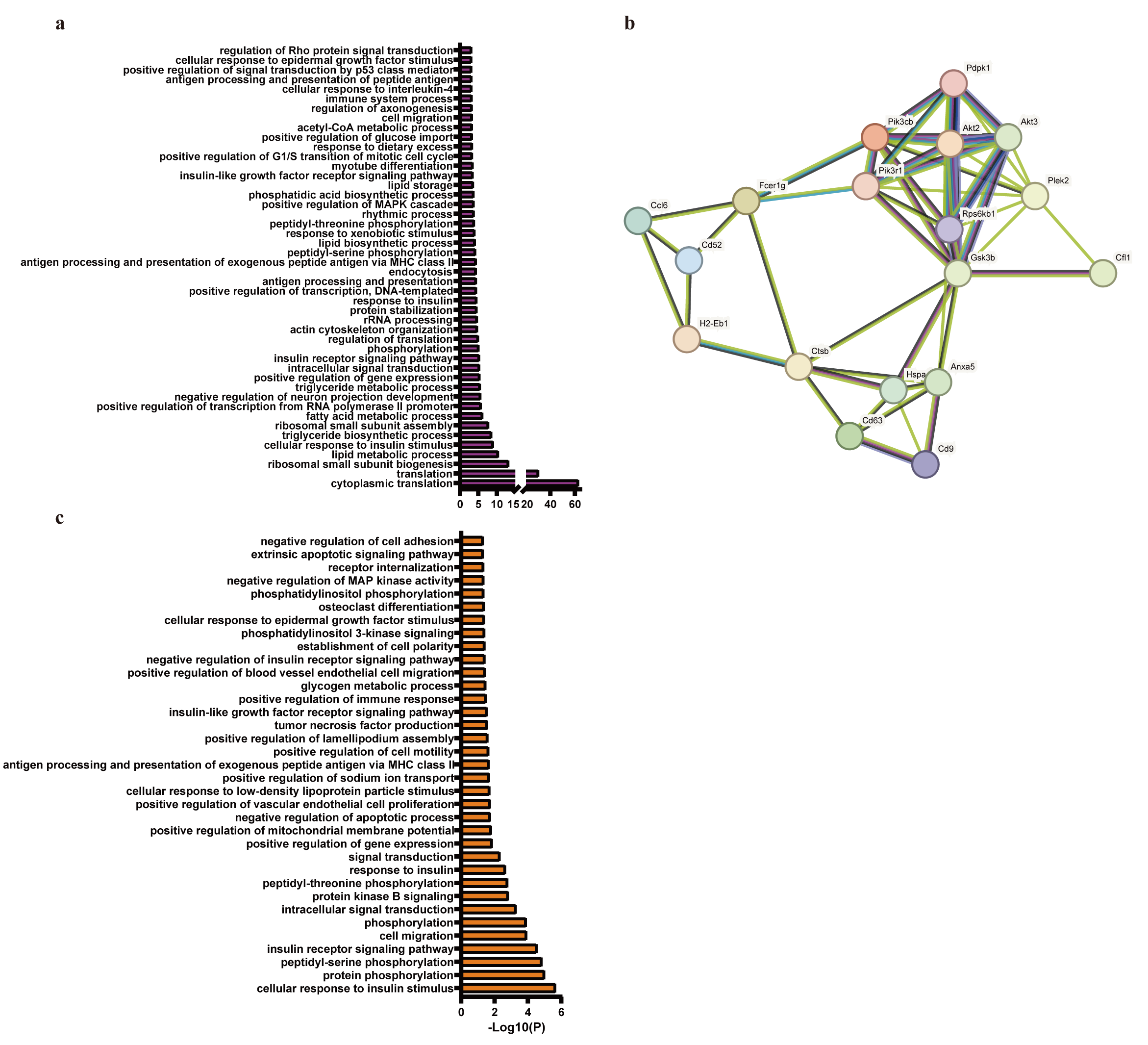

Supplement: Supplementary file 1 — Supplementary Information. [file 41598_2024_66039_MOESM1_ESM.zip › Figure S7.tif]
